# Leaf rust resistance in wheat and interpretation of the antifungal activity of silver and copper nanoparticles

**DOI:** 10.1038/s41598-025-91127-4

**Published:** 2025-03-19

**Authors:** Atwa A. Atwa, Shreen S. Ahmed, Gehan H. Abd El-Aziz, Mohamed A. Abou-Zeid, Reda I. Omara, Nourhan A. Atwa, Ashraf H. Fahmy

**Affiliations:** 1 Plant Protection Research Institute, Agriculture Research Center, Giza, 12619 Egypt; 2 Soils, Water and Environment Research Institute, ARC, Giza, 12619 Egypt; 3 Wheat Diseases Research Department, Plant Pathology Research Institute, ARC, Giza, 12619 Egypt; 4 Plant Genetic Transformation Department, Agricultural Genetic Engineering Research Institute, ARC, Giza, 12619 Egypt

**Keywords:** Defense mechanisms, Disease resistance, Molecular docking, Nanotechnology, Pathogen inhibition, Phytoalexins, Plant sciences, Planetary science

## Abstract

Wheat production is jeopardized by *Puccinia triticina*, the pathogen responsible for wheat leaf rust. This study assessed the impact of silver (Ag) and copper (Cu) nanoparticles (NPs) on the control of wheat leaf rust disease and the underlying mechanisms of disease resistance. The application of the two nanoparticles resulted in a reduction of spore germination and an extension of both incubation and latent periods. A common type of infection resulted in a reduction in both the length and width of pustules. It reduced receptivity value (number of pustule cm^2^) compared to untreated wheat plants by altering the physiological and biochemical responses of wheat plants and cell walls’ physical and mechanical strength. The application of Ag + Cu NPs stimulates the biosynthesis of defense-related molecules crucial for *P. triticina* inoculation and latent periods. Furthermore, molecular docking studies were conducted to assess the effects of Cu-chitosan nanoparticles (Ag & CuNp) and their mechanisms in disease management.

## Introduction

Wheat (*Triticum aestivum* L.) is the most important staple crop worldwide, cultivated on approximately 215 million hectares, with an annual global production exceeding 772 million tons (FAOSTAT, 2020). This cereal grain is crucial for food security, especially in developing countries, with demand projected to increase by 60% by 2050^1^. However, wheat production faces significant challenges, particularly from *Puccinia triticina,* the causative agent of leaf rust. This fungal pathogen represents a significant threat to wheat production, potentially decreasing yields by as much as 50% in susceptible varieties under epidemic conditions^2^. The constant emergence of new virulent races of *P. triticina* complicates the development and maintenance of resistant wheat varieties, highlighting the need for innovative and sustainable disease management strategies^3^.

Conventional methods to control wheat rust, such as fungicide use and resistance breeding, have demonstrated limited efficacy. Fungicides are extensively utilized; however, they present significant environmental and health risks. In addition, their excessive application contributes to the development of resistance to pathogens^4^. Similarly, the durability of genetic resistance in wheat is often compromised by the rapid evolution of pathogen virulence. These limitations have prompted the investigation of alternative strategies, such as the use of nanotechnology-based interventions^5^.

Nanotechnology offers a promising method for advancing sustainable agricultural practices. Ag and Cu NPs are significant due to their roles as antimicrobial agents and promoters of plant growth^6^. These nanoparticles exhibit distinct physicochemical properties, including increased surface-area-to-volume ratios and enhanced reactivity, which enhance their efficacy in inhibiting pathogen growth and inducing plant defense mechanisms^7^. Furthermore, Ag + Cu NPs have been shown to enhance the biosynthesis of defense-related molecules, strengthen cell wall integrity, and modulate physiological responses, thereby delaying disease onset and progression^8^.

Despite these advancements, the application of Ag + Cu NPs in controlling *P. triticina* in wheat remains underexplored. This study aims to address this gap by evaluating the antifungal activity of Ag and Cu NPs against wheat leaf rust under controlled greenhouse and field conditions.

The current study’s results are expected to advance our understanding of nanoparticle-mediated disease control, offering a novel and environmentally friendly strategy for managing wheat rust. This study integrates nanotechnology into traditional plant protection frameworks, contributing to global efforts to enhance wheat productivity and reduce the environmental impact of agricultural practices. The study specifically aims to evaluate the effects on critical disease parameters, such as spore germination, incubation and latent periods, pustule dimensions, and infection severity. Additionally, molecular docking analyses are utilized to elucidate the underlying mechanisms through which these nanoparticles affect pathogen biology and plant defense pathways.

## Materials and methods

This study was conducted in the leaf rust greenhouse at the Agricultural Research Center in Giza, Egypt. The experimental conditions included a temperature range of 20–22°C, a light/dark cycle of 14/10 h, and a relative humidity of 50–55%. Morocco, a susceptible variety, is cultivated in a greenhouse, with 10 grains per pot in plastic pots with a diameter of 10 cm and clay soil inside. We conducted the inoculation and incubation processes seven days post-planting, following the methods outlined in El-Orabey et al.^9^ and Atia et al.^10^.

To encourage the germination of spores and the spread of infection, the seedlings were gently rubbed with water between moistened fingers. Next, sterile spatulas were used to scrape out contaminated samples, which were placed in the seedlings and lightly sprayed with water. After being exposed to leaf rust spores for 24 h in damp chambers with 18–20˦ ◦C and 100% RH, the infected seedlings were then moved to benches in a greenhouse and kept there for 14 days at 20 ± 2 ◦C, 50–55% relative humidity, and 8000 Lux light intensity (14 h of light and 10 h of darkness). Data on rust appeared twelve days after pustules first appeared. After 12 days, rust data were recorded following pustule rupture. According to Johnston and Browder^11^, rust symptoms were classified according to the type of infection they represented. Resistance (= 0, 0,1 and 2) and susceptibility (= 3 and 4) denoted low and high infection types, respectively. The application techniques were a = spray 24 h before inoculation, B = spray 24 h after inoculation, and C = spray 24 h before and after. Ten milliliters per plant were sprayed with treatments of salicylic acid and Ag & Cu nanoparticles.

### Determination of the five resistance components at the seedling stage under favorable greenhouse conditions

Each pot received 10 wheat kernels in clay soil. Eight-day-old seedlings were uniformly inoculated with the urediniospores of the aggressive leaf rust race TTTS. Inoculation was performed as previously mentioned.

### The incubation period (IP)

Was measured by estimating the period (days) between the inoculation and the first appearance of a visible disease reaction on each wheat seedling under study.

### The latent period (LP)

Was estimated by counting the visible pustules. On marked. Leaves daily until no more pustules appeared, assessed for each genotype under study. As the time per day between inoculation and 50% of the pustule ruptured, according to the following equation. of Das et al.^12^.

$${\text{Latent period }} = \, \left[ {{\text{t}}_{{{1}.}} + \, \left( {{\text{F}}/{2 } - {\text{nt}}_{{1}} } \right). \, \left( {{\text{t}}_{{2}} - {\text{t}}_{{1}} } \right)} \right] \, / \, \left( {{\text{nt}}_{{2}} - {\text{nt}}_{{1}} } \right)$$

where F is the final number of uredinia, t_1_ is number. of days previous to 50% uredinia observed, t_2_ is the days after 50% uredinia observed, nt_1_ is the number of uredinia observed at t_1_, and nt_2_ is number of uredinia. observed at t_2_.

Pustule dimension (PS) was measured using a light microscope at 10× magnification. The pustules were fixed in a boiling solution of lactophenol and ethanol (1:2, v/v) for three minutes. The length (L) and width (W) of ten randomly selected pustules from each leaf were calculated using the formula from Broes^13^.

Pustule dimension (PS) was measured at 10× power magnification with a light microscope, and pustules were fixed for three minutes in a boiling solution of lactophenol and ethanol (1:2, v/v). Ten randomly selected pustules per leaf were measured for length (L) and width (W) using the Broes^13^ formula.

$${\text{Pustule dimension }} = \, \raise.5ex\hbox{$\scriptstyle 1$}\kern-.1em/ \kern-.15em\lower.25ex\hbox{$\scriptstyle 4$} \, \times . \, \pi {\text{ L }} \times {\text{ W Where}}: \, \pi . \, = { 3}.{14};{\text{ L is the length}};{\text{ W is the width of each pustule}}.$$

Receptivity (pustule length and pustule width) (number of uredinia/cm^2^). Was computed by dividing the total number of uredinia by the leaf area where uredinia were counted.

### Specimen preparation for SEM

The collected leaves were cut into approximately 3 × 3 mm squares and fixed for 24 h in a 3% glutaraldehyde solution in 0.05 M sodium cacodylate buffer at pH 6.8–7.2. After two rinses in the buffer, they were postfixed for 2 h in 2% osmium tetroxide and then dehydrated using a graded series of ethanol. Mounted on copper stubs, specimens underwent critical-point drying using carbon dioxide as a transition fluid. For specimens obtained at 12–96 hpi, Hughes & Rijkenberg’s^14^ leaf-fracture method was applied. The two fractured stubs were prepared for observation. Unfractured samples were taken at 6 and 144 hpi. The peeled epidermis and the remaining tissue on the stubs were coated with gold and palladium in a Sputter coater. The samples were analyzed using a Jeol JSM-5200 scanning electron microscope at accelerating 8- or 10 kV voltages. Specimens collected 6 and 144 h post-infection (hpi) were examined externally only. Some samples were freeze-fractured using an EM Scope SP 2000 cryo unit and viewed at 8 kV, while others were analyzed without fracturing. The infection structures were counted directly from the screen.

### Synthesis and characterization of copper nanoparticles

Cu O nanoparticles were created according to Phiwdang et al.^15^ by precipitating copper nitrate (Cu NO_3_)_2_.3H_2_O) and copper chloride (CuCl_2_). To create a 0.1 M concentration, each precursor was first dissolved in 100 ml of deionized water. The 0.1 M NaOH solution was gradually added while vigorously stirring until the pH reached 14. After obtaining black precipitates, deionized water, and absolute ethanol were washed repeatedly until the pH reached 7. The cleaned precipitates were then dried for 16 h at 80 °C. Ultimately, the precursors underwent a 4-h calcination at 500 °C while being examined using X-ray diffractometry (XRD). By using a scanning electron microscope, morphology was observed (SEM).

### Synthesis and characterization of silver nanoparticles

Silver nanoparticles were synthesized using a precipitation method similar to that described by Phiwdang et al.^15^. To create the nanoparticles, 100 mL of deionized water was mixed with 0.1 M copper nitrate (Cu (NO_3_)_2_·3H_2_O) and 0.1 M copper chloride (CuCl2). A 0.1 M sodium hydroxide (NaOH) solution was added gradually while stirring vigorously until the pH reached 14. The resulting black precipitates were washed with deionized water and absolute ethanol multiple times until the pH reached 7. The cleaned precipitates were dried at 80°C for 16 h, followed by a 4-h calcination at 500°C. The nanoparticles obtained were characterized using X-ray diffractometry (XRD) and scanning electron microscopy (SEM) to analyze their morphology and structure.

### Nanoparticle characterization by transmission electron microscopy (TEM)

Using a 400-mesh Formvar^®^ carbon-coated copper grid, an FEI Spirit TEM (Hillsboro, USA) operating at 120kV was used to generate TEM micrographs. Cu and Ag NPs were prepared according to^16^ by vortexing 2.0 L of the sonicated colloidal solution onto the grid, re-sustaining the sample with a 10-ml disposable pipet, and creating EM grids (carbon-coated 400-mesh copper grids) directly on the specimen, specimen-side up in the specimen petri dish.

### Statistical analysis

Three repetitions of a randomized complete block design (RCBD) were employed. The data were statistically analyzed using analysis of variance (ANOVA) with SPSS software version 22.0 (SPSS Inc., Chicago, IL, USA).

## Results

The effect of the different application methods of two nanoparticles on leaf rust disease components.

### Greenhouse studies

Incubation, latent period, and infection types: the data presented in Table 1 demonstrates how three treatments and four application methods influenced leaf rust, race TTTS, during the seedling stage’s incubation period, latent period, and infection types. The results indicate that crown fungicide and silver (Ag), copper (Cu), and their combined nanoparticles (NPs) demonstrated a significant impact on the incubation and latent periods of *Puccinia triticina* infection and the resulting infection types. Table 1 provides a detailed summary of these effects across different treatments and application methods.

**Table 1:** The effect of Ag, Cu NPs and their combination, as well as four application methods on incubation, latent periods and infection type of wheat leaf rust disease.

Treatment	Incubation, Latent periods, Infection type/application											
	Incubation period/day				Latent period/day				Infection type			
	A*	B*	C*	D*	A	B	C	D	A	B	C	D
Ag NPs	10.00	11.00	12.6	14.3	14.6	15.0	17.6	18.6	2 +	2	1 +	1 +
Cu NPs	8.0	9.6	10.3	12.0	13.0	14.0	15.3	16.0	3	2 +	2	2
Ag + Cu NPs	11.3	12.6	13.0	15.3	15.0	17.3	18.0	19.0	2	2-	1 +	1
Crown (Fungicide)	0.00	0.00	0.00	0.00	0.00	0.00	0.00	0.00	0	0	0	0
Control	6.3	6.3	6.3	6.3	10.0	10.0	12.0	10.0	4	4	4	4
LSD_0.05_	1.01	1.12	1.02	0.95	0.64	0.75	0.71	0.62	-	-	-	-

A* = seed treatment, B* = seed treatment and spray before inoculation by 24 h, C* = spray before inoculation by 24 h, D* = spray after inoculation by 24 h.

The combination of Ag + Cu NPs demonstrated the most significant effect among the treatments, particularly when administered 24 h after inoculation (Method D). The incubation period increased to 15.3 days, while the latent period extended to 19.0 days, compared to the untreated control. It recorded an incubation period of 6.3 days and a latent period of 10.0 days. The observed delay in disease progression suggests a significant inhibitory effect of the combined nanoparticles on the early stages of pathogen development (Table 2).

**Table 2:** The effect of Ag, Cu NPs and their combination, as well as four application methods on the development of pustules in *P. tritici*.

Treatment	Pustules development/application											
	Number of pustules				Pustule length μ_m_				Pustule width μ_m_			
	A*	B*	C*	D*	A	B	C	D	A	B	C	D
Ag NPs	45.3	41.2	36.3	10.5	287.5	254.2	192.4	162.3	144.0	135.3	125.2	102.2
Cu NPs	49.3	46.2	40.4	20.3	302.2	277.9	210.3	188.2	155.3	148.8	140.4	122.4
Ag + Cu NPs	41.3	36.5	30.4	8.3	257.3	212.4	182.2	144.6	130.0	127.5	117.7	97.3
Crown (fungicide)	0.0	0.0	0.0	0.0	0.0	0.0	0.0	0.0	0.0	0.0	0.0	0.0
Control	72.4	69.2	68.0	70.2	412.2	420.4	426.8	428.2	221.0	226.4	231.5	234.2
LSD_0.05_	3.42	4.53	4.87	5.21	9.32	9.45	10.23	9.43	7.56	8.43	10.21	9.31

A* = seed treatment, B* = seed treatment and spray before inoculation by 24 h, C* = spray before inoculation by 24 h, D* = spray after inoculation by 24 h.

Infection-type assessments further validated the efficacy of Ag + Cu NPs. The treatment resulted in an infection-type score of “1” under Method D, indicating minimal infection. In contrast, the controls demonstrated a score of “4,” which indicates a severe infection. The crown fungicide demonstrated complete suppression of infection (score = 0), serving as a standard for assessing the efficacy of nanoparticle treatments. The findings indicate that Ag + Cu NPs can effectively reduce disease severity by hindering pathogen establishment and proliferation (Table 1).

### Infection type and pustule development

**Fig. 1:**
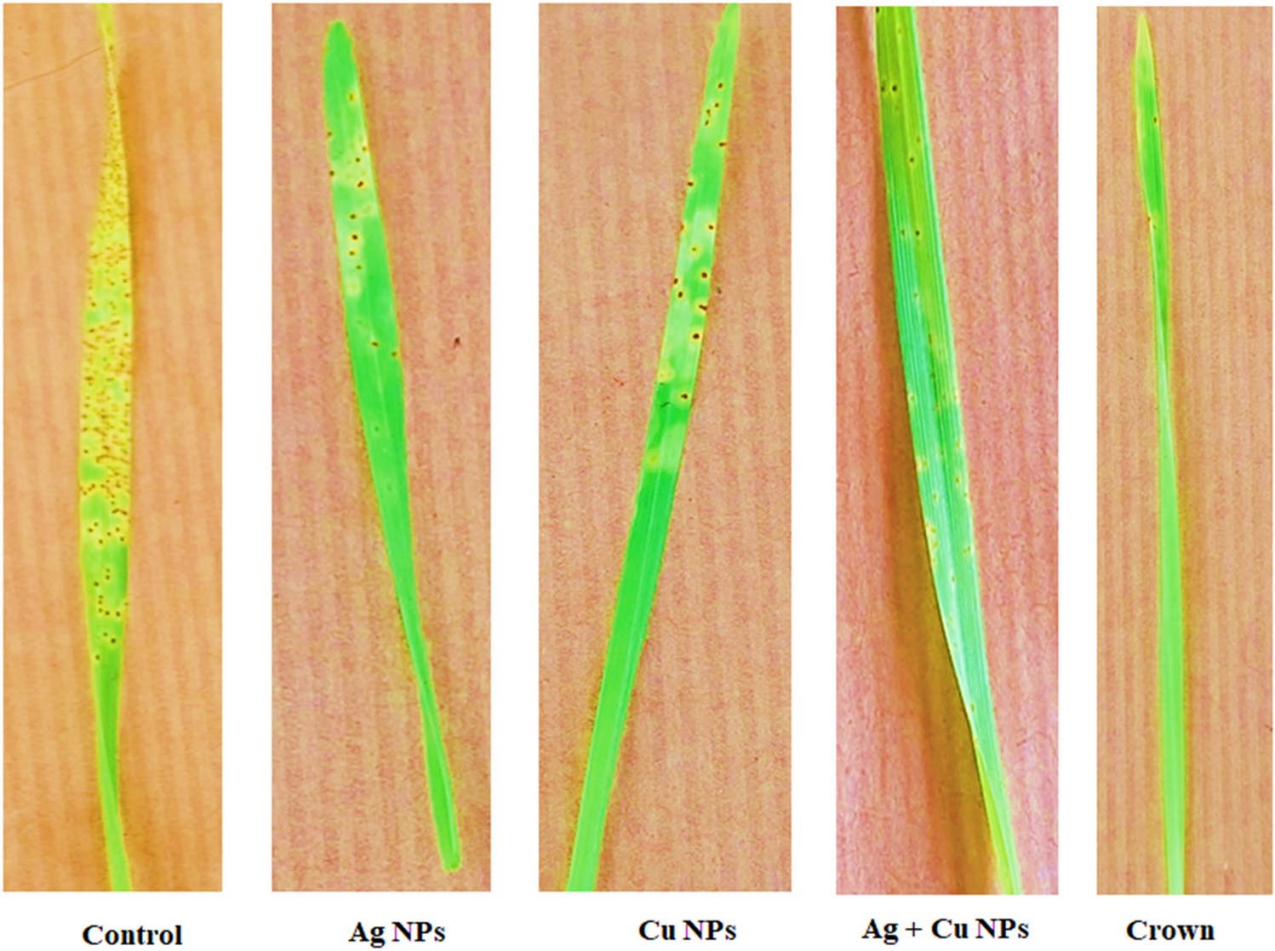
Infection type of three treatments sprayed 24 h after inoculation. Number of pustules per cm^2^, the length and width of the pustules.

Figure 1 presents the types of infections and the characteristics of pustules associated with various treatments. Plants treated with Ag + Cu NPs showed significantly reduced pustule numbers, as well as reductions in both length and width, compared to the control group. Method D resulted in a reduction of pustules per cm^2^ to 8.3 compared to 70.2 pustules per cm^2^ in the untreated control group. The dimensions of the pustules (length and width) were significantly decreased to 144.6 µm and 97.3 µm, respectively, in contrast to the control measurements of 428.2 µm and 234.2 µm. The reductions demonstrate the significant antifungal efficacy of the nanoparticles in inhibiting pathogen growth.

The infection-type ratings presented in Fig. 1 align closely with the data in Table 1, which further validates the findings. The treatment with Ag + Cu NPs exhibited enhanced efficacy in inhibiting disease progression, indicating its potential as a sustainable alternative to conventional fungicides.

### Laboratory studies

**Fig. 2:**
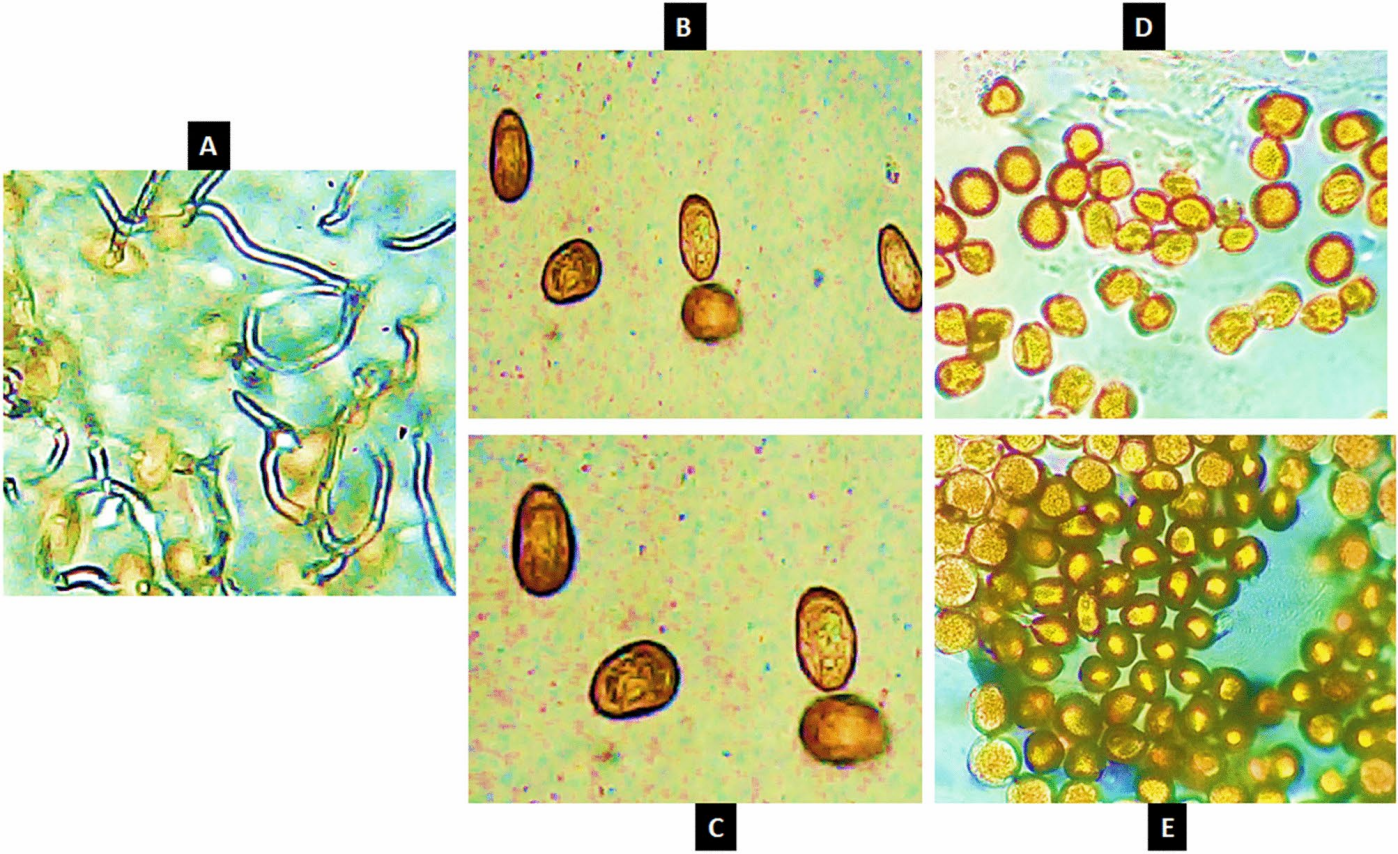
effect of Ag (**A**), Cu (**B**), Ag + Cu NPs (**D**) and crown fungicide (**E**) on *P. triticina* urediniospore germination compared to control (**A**).

The effect of treatments on the germination of urediniospores: The impact of Ag, Cu, Ag + Cu NPs, and crown fungicide on the germination of *P. triticina* urediniospores was assessed using a water-agar medium (Fig. 2). The results indicate a significant difference in germination ability across all treatments. Ag + Cu NPs demonstrated superior efficacy in inhibiting germination compared to Ag NPs and the crown fungicide, highlighting its potential as a viable alternative in managing urediniospore populations (Fig. 2D, B).

### Electron microscope analysis

**Fig. 3:**
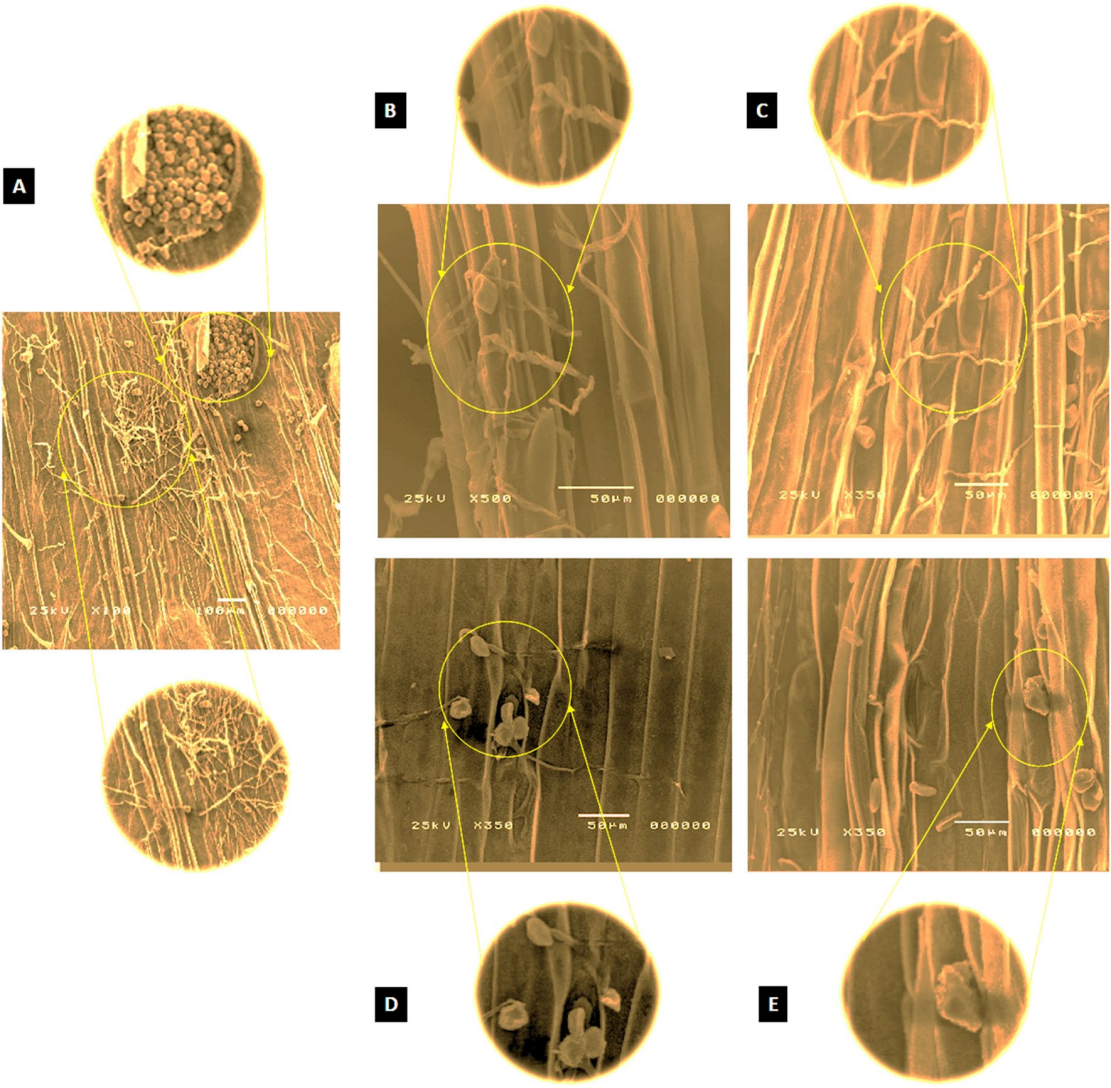
Scanning electron microscope observations of spores and mycelia of *P. tritici* taken from wheat leaves of some treatments show (**A**) Control with untreated spores and mycelium. (**B–E**) treated with Ag, Cu, Ag + Cu NPs, and crown, showing collapsed mycelia and spores, respectively.

Scanning electron microscope (SEM) analysis of fungal structures collected from wheat leaves at the seedling stage revealed notable anomalies and alterations. Figure 3 depicts the morphological changes in Puccinia triticina spores and mycelia across various treatments. Panel 3A, depicting the untreated control, displays intact and healthy fungal structures characterized by well-formed urediniospores and robust mycelia, which indicate active fungal growth without any intervention. Panels 3B and 3C demonstrate the effects of Ag and Cu NPs individually, showing significant structural damage characterized by deformation and partial collapse of urediniospores and mycelia. The 3D panel, illustrating the treatment with combined Ag + Cu NPs, demonstrates the most significant morphological disruptions. The findings indicate substantial plasmolysis, total spore collapse, and fungal mycelia disintegration, demonstrating the combined nanoparticles’ synergistic effects. Panel 3E, representing the fungicide treatment (Crown), exhibits moderate damage to fungal structures, including signs of deformation and compromised integrity.

The observed differences among treatments highlight the enhanced antifungal efficacy of the combined Ag + Cu NPs treatment, surpassing both individual nanoparticle applications and the traditional fungicide. The findings underscore the efficacy of nanoparticles, especially in combined formulations, as a sustainable method for managing wheat rust diseases.

### Total phenol and electrolyte leakage

**Fig. 4:**
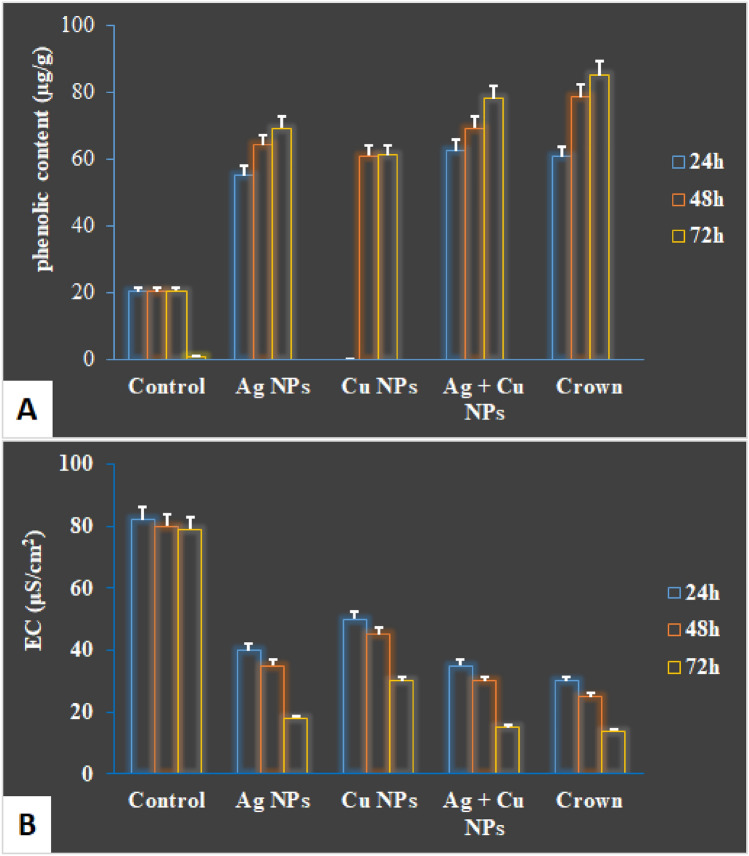
Effect of some treatments on phenolic content (**A**) and electrolyte leakage (**B**) at 24, 48 and 72 h against wheat leaf rust.

To elucidate the role of total phenols concerning *P. triticina* infection, levels were measured in three treatments along with crown fungicide (see Fig. 4A). Results showed that all treatments significantly increased total phenol levels when compared to both crown and untreated plants, particularly the Ag + Cu NPs treatment. This increase in phenolic compounds likely contributes to enhanced resistance against the pathogen. Moreover, all treatments, especially Ag + Cu NPs, significantly reduced electrolyte leakage in wheat plants affected by leaf rust, as indicated in Fig. 4B. This suggests improved cellular integrity and stress response in treated plants.

### Enzyme activities

**Fig. 5:**
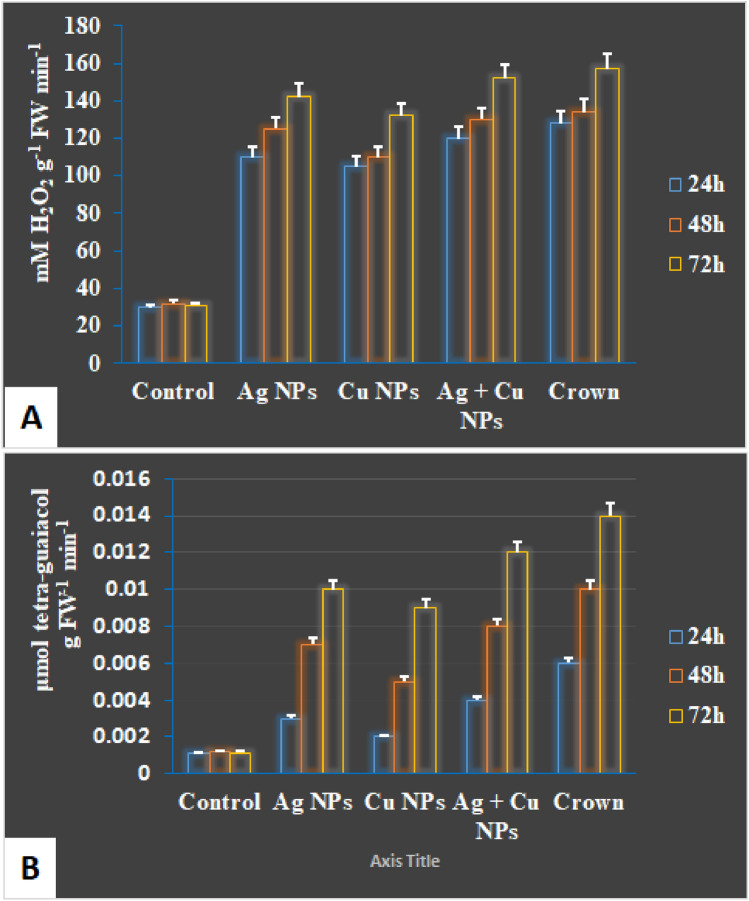
The activities of the antioxidant defense enzymes catalase (**A**) and peroxidase (**B**) at 24, 48, and 72 h of some treatments were tested against wheat leaf rust.

The activities of two key antioxidant enzymes, catalase (CAT) and peroxidase (POX), were evaluated to determine the role of oxidative stress management in nanoparticle-treated plants. Figure 5 shows that all treatments significantly increased CAT and POX activities compared to the untreated control, with Ag + Cu NPs inducing the highest enzyme activity levels. These enzymes play critical roles in scavenging reactive oxygen species (ROS), thereby protecting plants from oxidative damage caused by pathogen infection.

CAT activity (Fig. 5A) exhibited a consistent increase across all treatments, peaking at 72 h post-treatment. This enhancement indicates improved ROS detoxification, preventing cellular damage, and supports systemic acquired resistance (SAR). Similarly, POX activity (Fig. 5B) was significantly higher in nanoparticle-treated plants, with Ag + Cu NPs exhibiting the most pronounced effect. POX plays a role in lignin biosynthesis and the cross-linking of cell wall components, thereby enhancing the physical barrier against pathogen invasion.

**Fig. 6:**
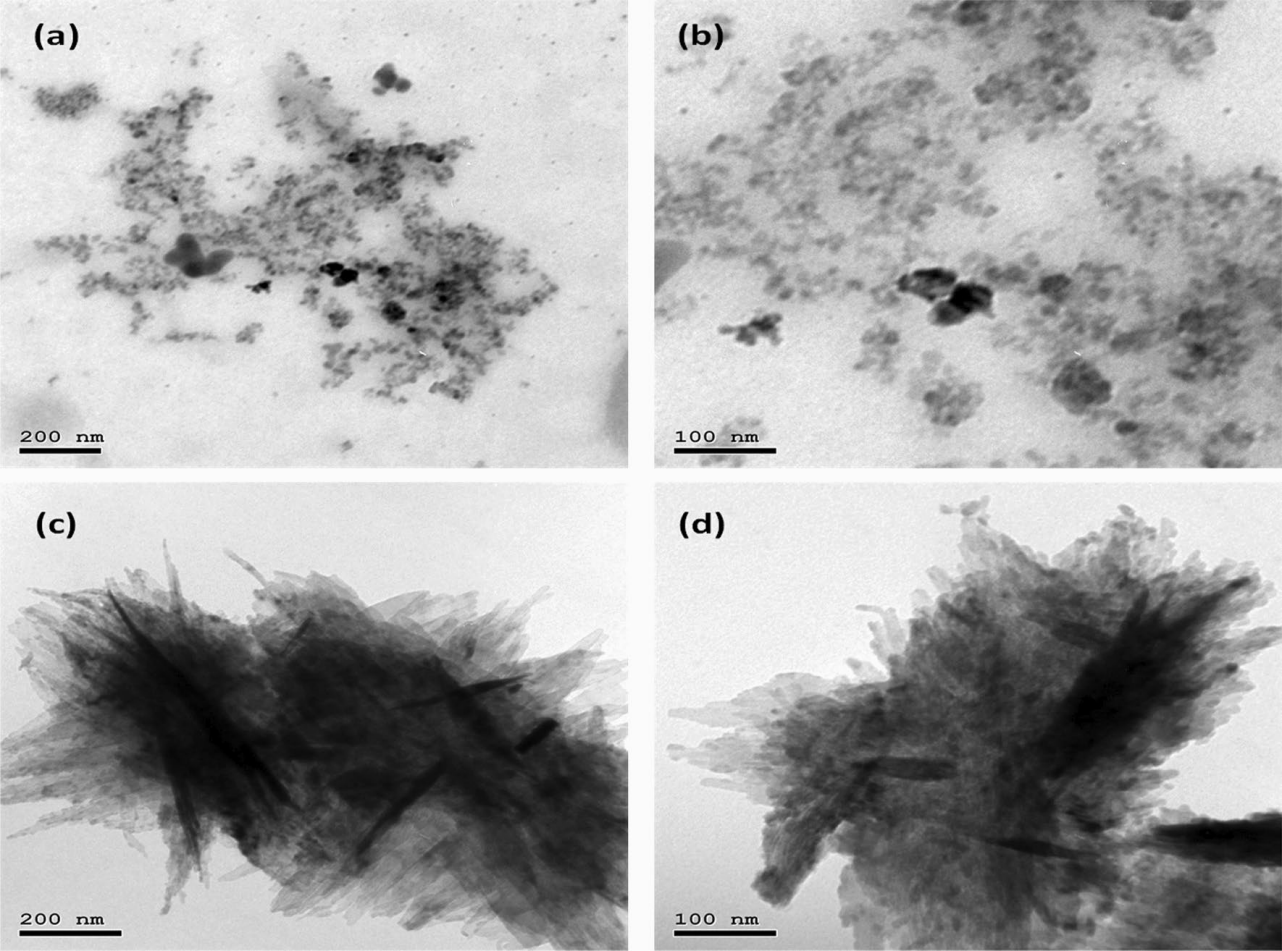
(**a,b**) The low and high magnification TEM of Ag sample, and c and d the low and high magnification TEM of Cu sample.

Figure 6 presents the TEM images of Ag and Cu NPs at low and high magnifications. The Ag nanoparticles appeared as spherical structures with uniform sizes, whereas the Cu nanoparticles exhibited a needle-like morphology. The average size of the nanoparticles was consistent with the expected range for efficient biological activity. The well-defined and consistent morphologies of these nanoparticles are crucial for their interactions with fungal cells and their ability to induce plant defense mechanisms.

The Transmission Electron Microscopy (TEM) images show that the Ag sample consisted of small spheres with a uniform structure, as seen in Fig. 6a, b. Conversely, the Cu images revealed that the synthesized nanoparticles exhibited a needle-like structure (see Fig. 6c, d), further confirming the successful fabrication of nanoparticles.

**Fig. 7:**
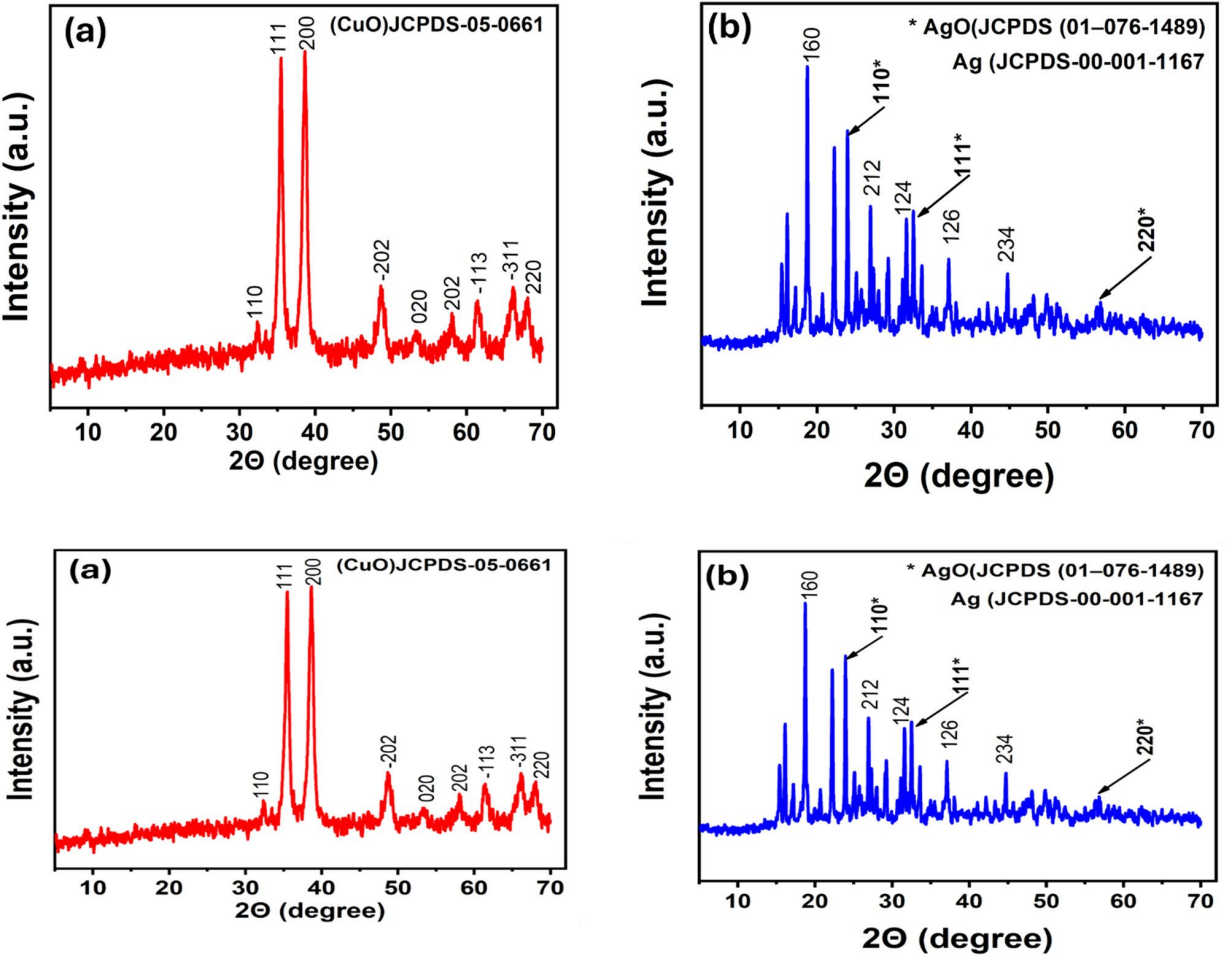
X-ray diffraction (XRD) of Cu and Ag nanoparticles.

Furthermore, X-ray diffraction (XRD) analysis was employed to identify the crystalline nature and phases of the synthesized compounds. The XRD patterns analyzed corresponded to the following 20 values: 32.35°, 35.62°, 38.69°, 48.72°, 53.49°, 58.33°, 61.57°, 66.2°, and 66.31°, corresponding to the (110), (111), (200), (−202), (020), (202), (−113), (−311), and (022) reflection lines of monoclinic Cu nanoparticles (JCPDS-05-0661). This indicates the crystalline nature of the synthesized Cu nanoparticles (Fig. 7).

The XRD analysis of the Ag-prepared sample depicted in Fig. 7 identified peaks at 18.90°, 27.62°, 31.44°, 37.84°, and 45.95°, corresponding to the (160), (212), (124), (126), and (234) planes of silver, as per JCPDS-00-001-1167. Diffraction angles of 24.9°, 32.2°, and 54.72° correspond to the (110), (111), and (220) planes, respectively, thereby confirming the properties of AgO nanoparticles as outlined in JCPDS (01-076-1489).

## Discussion

Leaf rust is a frequent disease in every region where wheat is grown. Field observations revealed that it manifests yearly to various degrees in various regions^9^. The susceptibility of each variety to this disease affects how severe the infection is,therefore, chemical resistance must be employed when planting highly vulnerable varieties to minimize the potential losses^2^. To prevent widespread infection of the vulnerable genotypes, this can also be accomplished by creating rust-resistant genotypes or by successfully manipulating the existing genotypes across the nation Omar et al. (2021) and Thabet et al.^17^.

As a result, chemical fumigants should be utilized as the second line of defense in pest control. These chemical fungicides contribute to environmental pollution and pose risks to human health, particularly for individuals lacking awareness of necessary precautions when handling pesticides^4^. The study employed various safe substances, including Ag and Cu NPs, to combat this disease^5^.

The impact of these substances on the germination of urediniospores and the progression of disease was evaluated. The treatment significantly inhibited urediniospore germination and disease progression by decreasing the incubation period, latent period, infection types, number of pustules, and both the length and width of pustules in comparison to untreated plants^8^. This aligns with the findings of Elmer and White^7^ and Elmer et al.^18^, who discovered that Cu2O NPs had great potential for suppressing soil-borne fungi such as *F. solani* and *F. oxysporum.* According to Consolo et al.^5^, Ag and Cu2ONPs significantly inhibited the growth of *A. alternata* and *P. oryzae* mycelia at dose-dependent concentrations. Induction of systemic resistance (ISR) as a primary mechanism of plant response may have a favorable effect on both incubation and latent period times (Loeffler et al. 1986). Additionally, using Ag and Cu NPs may improve the biosynthesis of defense-associated molecules crucial in postponing *P. triticina* inoculation and the latent period. This occurs by modifying the physiological and biochemical responses of wheat plants as well as the physical and mechanical strength of cell walls^8,19^ (Mohdly et al. 2024).

Systemic acquired resistance was induced in wheat plants. NPs had no impact on the formation of infection structures induced by the brown rust pathogen *Puccinia triticina* on the surface of the plant. It exerts an inhibitory effect on mycelium development in plant tissues. The impact of Ag and Cu nanoparticles on pathogenesis varied depending on the infection status of the plant, specifically whether it was infected with a virulent or avirulent fungal clone. The inhibition of mycelium development in the virulent clone was associated with increased hypersensitivity reaction (HR). The effect on virulent clones primarily entailed preventing infection and forming infection structures, leading to alterations in cell shape, which resulted in a “plant fungicide-like effect”^20^. Studies by Fiori et al.^21^ and Farid et al.^22^ indicate the sensitivity of the target fungus. The origin of a medicinal plant influences its efficacy against various species of pathogenic fungi. The results demonstrate that essential Ag and Cu NPs, including frankincense oil (Boswellia carteri Birdw), can inhibit the growth of *P. triticina* mycelium and spore germination. This indicates that Ag and Cu nanoparticles may serve as alternatives to synthetic fungicides in managing pathogenic fungi^23^. 

All treatments significantly increased total phenol levels, with the Ag + Cu NPs treatment demonstrating the most pronounced effect on the plant’s metabolism. All treatments significantly reduced electrolyte leakage in wheat-treated plants relative to untreated plants. The pathogen’s effects on the plasma membrane in control plants, which enhance membrane permeability, may account for these outcomes. Ag and Cu nanoparticles exhibited superior performance in reducing electrolyte leakage compared to untreated plants. Electrolyte leakage (EL) occurs in response to various plant stressors, including salinity, pathogen attack, drought, heavy metals, hyperthermia, and hypothermia, serving as a defensive mechanism^24^ (Omar et al. 2021). The activities of catalase (CAT) and peroxidase (POX) significantly increased in treated plants, particularly at 72 h. CAT, an oxidative enzyme, plays a vital role in enhancing host tolerance to plant pathogen control (Liau & Lin, 2008). Enhanced catalase activity reduced cytosolic hydrogen peroxide levels, resulting in toxic conditions that hindered pathogen growth. This process also served as a secondary signal for the expression of defense genes and the activation of systemic acquired resistance^25^. Peroxidase is the first enzyme that exhibits altered activity in response to environmental stress. Additionally, it is known that modifications to this antioxidant enzyme play a direct role in activating plant defensive responses.

The use of Ag and Cu NPs in agriculture raises significant environmental concerns, particularly regarding their potential effects on soil health and the risk of nanoparticle accumulation over time. Extended exposure to nanoparticles has prompted concerns regarding soil toxicity, potentially disrupting beneficial microbial communities critical for plant health and nutrient cycling. Recent studies indicate that Ag and Cu NPs have a negligible residual effect on soil ecosystems when administered in controlled dosages. A study by Consolo et al.^5^ indicated that the application of Cu NPs at moderate concentrations did not result in significant soil accumulation, thereby reducing environmental risks. Elmer and White^7^ illustrated that Cu NPs degrade in soil environments over time, leading to a limited long-term impact on soil composition and microbial diversity. These findings highlight that the controlled and targeted use of Ag and Cu NPs can reduce potential environmental risks, positioning them as a sustainable alternative to conventional fungicides while preserving soil health.

### Comparative cost and scalability

The initial costs of Ag and Cu NPs fabrication may exceed those of conventional fungicides; however, evidence suggests that their long-term efficacy and reduced application frequency could offset or lower overall agricultural costs. Elsharkawy et al.^8^ showed that a single application of Ag and Cu Nps significantly diminished pathogen activity and postponed disease progression in wheat, thus extending the interval between necessary treatments. Research shows that nanoparticles exhibit enhanced effectiveness at lower concentrations, leading to significant reductions in the amount of product required for adequate crop protection (Menzies and Belanger 1996). The properties of nanoparticles, along with their prolonged action, despite high initial production costs, and the long-term economic benefits make nanoparticles a viable and cost-effective option for extensive agricultural application. The application’s scalability, coupled with diminished environmental risks, renders Ag and Cu NPs viable agents in sustainable agriculture.

The study further contributes to the wider understanding of nanoparticle-based interventions in agriculture, hence offering a new strategy for improving crop protection with minimal environmental and health risks. The superior performance of Ag and Cu NPs in inhibiting fungal activity and enhancing plant resistance mechanisms indicates their potential to revolutionize traditional approaches to disease control. In other words, nanoparticles reduce not only the challenge of resistance developed by fungicides but also provide a venue to reduce dependence on chemical treatment in an agricultural world that has to become increasingly sustainable.

Further research is required to build upon these findings, considering the long-term environmental and ecological effects of nanoparticles, especially interaction with soil microbiota and plant health across successive cultivation cycles. Field-scale applications under diverse climatic and agronomic conditions are also necessary to establish the efficacy and scalability of the nanoparticle treatments. Such efforts will enable Ag and Cu NPs to express their full potential in wheat production and crop protection toward sustainable development, thus finding a place in more extensive agricultural practice.

## Conclusion

The present study enlightens the massive potential of Ag and Cu NPs against wheat leaf rust caused by *Puccinia triticina*. In this context, spore germination was reduced with the application of nanoparticles, especially in a combined way, while the main disease parameters such as incubation and latent periods, infection severity, and pustule dimensions were modified. These results highlight the promising role of Ag and Cu NPs as new, environmentally sustainable tools for disease management in wheat crops. Such nanoparticles enhance the physiological and biochemical defenses of wheat plants, contributing to the delay in the onset of the disease and the mitigation of its development, thus offering a solid alternative to traditional chemical fungicides.

## Data Availability

All data generated or analyzed during this study are included in this published article.
